# Supplementary material for: Oral Health and Frailty in Community-Dwelling Older Adults in the Northern Netherlands: A Cross-Sectional Study
Source: Int J Environ Res Public Health. 2022 Jun 23;19(13):7654. doi: 10.3390/ijerph19137654 (PMC9265776; doi:10.3390/ijerph19137654)
Supplement: Supplementary file 1 [file ijerph-19-07654-s001.zip › Supplement Table S1.pdf]

Online Supplement Table S1 Groningen Frailty Indicator (GFI) questionnaire items English version

Item	Question
Can you perform the following tasks without assistance from another person?	
**1**	Grocery shopping
**2**	Walk outside house (around house or to neighbour)
**3**	Getting (un)dressed
**4**	Visiting restroom
**5**	Do you encounter problems in daily life because of impaired vision?
**6**	Do you encounter problems in daily life because of impaired hearing?
**7**	Did you lost a lot of weight unintentionally in the past 6 months? (6kg in 6 months or 3kg in 3 months)
**8**	Do you use 4 or more different types of medication?
**9**	Do you have any complaints with your memory (or are you diagnosed with dementia)?
**10**	Do you ever experience emptiness around yourself? (e.g. you feel so sad that you have no interests in your surroundings. Or if someone you love no longer love you, how do you feel?)
**11**	Do you ever miss the presence of other people around you? Or do you miss anyone you love?
**12**	Do you ever feel left alone?
**13**	Have you felt feeling down or depressed lately?
**14**	Have you felt nervous of anxious lately?
**15**	How would you rate your own physical fitness?<br>(0-10; 0 is very bad, 10 is very good) 0-6 = 1 7-10=0
