# Supplementary material for: Oral Health and Frailty in Community-Dwelling Older Adults in the Northern Netherlands: A Cross-Sectional Study
Source: Int J Environ Res Public Health. 2022 Jun 23;19(13):7654. doi: 10.3390/ijerph19137654 (PMC9265776; doi:10.3390/ijerph19137654)
Supplement: Supplementary file 1 [file ijerph-19-07654-s001.zip › Supplement Table S2.pdf]

Online Supplement Table S2 Oral Health Impact Profile-14 questionnaire items, English version

Items	Question
1	Have you had trouble pronouncing certain words because of problems with your teeth, mouth or dentures?
2	Have you felt that your sense of taste has worsened because of problems with your teeth, mouth or dentures?
3	Have you had pain aching in your mouth?
4	Have you found it uncomfortable to eat any foods because of problems with your teeth, mouth or dentures?
5	Have you been self-conscious because of your teeth, mouth or dentures?
6	Have you felt tense because of your teeth, mouth or dentures?
7	Has your diet been unsatisfactory because of your teeth, mouth or dentures?
8	Have you had to interrupt meals because of problems with your teeth, mouth or dentures?
9	Have you found it difficult to relax because of problems with your teeth, mouth or dentures?
10	Have you been a bit embarrassed because of problems with your teeth, mouth or dentures?
11	Have you been a bit irritable with other people because of problems with your teeth, mouth or dentures?
12	Have you had difficulty doing your usual jobs because of problems with your teeth, mouth or dentures?
13	Have you felt that life in general was less satisfying because of problems with your teeth, mouth or dentures? Have you been totally unable to function
14	Have you been totally unable to function because of your problems with your teeth, mouth or dentures?
